# Investigation of the Influence of Fused Deposition Modeling 3D Printing Process Parameters on Tensile Properties of Polylactic Acid Parts Using the Taguchi Method

**DOI:** 10.3390/ma17235951

**Published:** 2024-12-05

**Authors:** Getu Koro Megersa, Wojciech Sitek, Agnieszka J. Nowak, Neven Tomašić

**Affiliations:** 1 Scientific and Didactic Laboratory of Nanotechnology and Materials Technologies, Silesian University of Technology, 44-100 Gliwice, Poland; agnieszka.j.nowak@polsl.pl; 2 Department of Materials Science and Engineering, Faculty of Engineering, University of Rijeka, Vukovarska 58, 51000 Rijeka, Croatia; neven.tomasic@uniri.hr

**Keywords:** Fused Deposition Modeling, tensile properties, Polylactic Acid, Taguchi method

## Abstract

Despite Fused Deposition Modeling (FDM) being an economical 3D printing method known for its material versatility and ease of use, the mechanical performance of FDM-produced components is significantly influenced by process parameter settings. This study investigated the effects of the layer thickness, raster angle, build orientation, and extrusion temperature on the ultimate tensile strength (UTS) and elastic modulus of Polylactic Acid (PLA) specimens using Taguchi methods, with significance analyzed through analysis of variance (ANOVA). The results indicated that the build orientation is the primary factor affecting both the UTS and elastic modulus, with a flat orientation yielding the best performance. ANOVA showed that the build orientation, raster angle, and extrusion temperature significantly influence the UTS, with the build orientation contributing 98.16%. For the elastic modulus, the build orientation and raster angle were significant, contributing 94.83% and 1.76%, respectively. The optimal parameters were a 0.16 mm layer thickness, flat build orientation, 30°/−60° raster angle, and 200 °C extrusion temperature, resulting in predicted UTS and elastic modulus values with error percentages of 4.33% and 2.74%, respectively, compared to experimental values. The regression model demonstrated high predictive accuracy, with R-squared values of 99.71% for the UTS and 99.52% for the elastic modulus.

## 1. Introduction

Rising demand for customized, lightweight, and complexly shaped products, coupled with the need to reduce material waste, production cycles, and manufacturing time, has led to the widespread adoption of direct manufacturing technologies from digital designs, known as additive fabrication or three-dimensional (3D) printing [1]. 3D printing’s flexibility and industry integration have expanded its use in sectors such as automotive, aerospace, healthcare, food, fashion, and construction [2,3]. Fused Deposition Modeling (FDM) is a cost-effective 3D printing technique that fabricates objects by extruding heated thermoplastic material layer by layer. Each layer fuses to the previous one, guided by G-code instructions derived from digital 3D models [4,5]. Polylactic Acid (PLA) is a widely used thermoplastic in FDM 3D printing, known for its low melting point, good strength, biodegradability, non-toxicity, and dimensional stability [6,7]. Although FDM technology offers affordability, material versatility, and ease of use, it faces challenges due to the impact of process parameters on the mechanical properties of printed parts, leading to weaker performance compared to parts made by conventional methods [8,9,10].

Many researchers have investigated the effect of FDM process parameters on the mechanical properties of PLA specimens fabricated using 3D printing technology. Torres et al. [11] investigated PLA specimens in flat, upright, and on-edge orientations, varying parameters such as the speed, temperature, infill direction, perimeter, and infill density. They found that on-edge samples had the lowest risk of failure, with infill density being the primary factor influencing the tensile strength and modulus across all orientations. Chacon et al. [4] studied the tensile and flexural strengths of FDM PLA specimens, varying the feed rate, build orientation, and layer thickness. They found that a higher layer thickness and lower feed rate improved tensile strength in the upright orientation, while a lower thickness and higher feed rate benefited flat and on-edge orientations. Upright specimens had the lowest tensile strength overall. Alafaghani et al. [12] applied the Taguchi method to assess the tensile strength and dimensional accuracy of PLA by varying the fill density, pattern, extrusion temperature, and layer height. They found that a lower extrusion temperature, infill density, and layer height, along with a hexagonal pattern, improved dimensional accuracy. The highest tensile strength was achieved with a triangular pattern at 100% density, 210 °C temperature, and 0.3 mm layer thickness.

Rajpurohit et al. [13] studied the impact of the raster angle, layer thickness, and raster width on tensile strength. They found that a lower layer thickness and wider raster width increased tensile strength. The highest tensile strength was achieved at a 0° raster angle, while the lowest was observed at a 90° raster angle. In the studies by Yao et al. [14] and Zhao et al. [15], both theoretical and experimental approaches showed that smaller printing angles and thicker layers reduced tensile strength in PLA, with Zhao et al. also noting a decrease in Young’s modulus [15]. Chadha et al. [16] studied the effect of layer thickness, infill pattern, and bed temperature on the tensile and flexural strengths. They found that increasing the bed temperature initially improved both strengths but eventually led to a decrease. Thicker primary layers enhanced both strengths, while triangular and honeycomb infill patterns exhibited the highest tensile and flexural strength. Both studies by N. H. Patadiya et al. [17] and Domenico Corapi et al. [18] demonstrated that the build orientation significantly impacts the tensile strength of fabricated PLA samples. Horizontally printed specimens (with rods arranged parallel to the applied load) exhibited higher tensile strength compared to those printed on-edge or vertically. Wang et al. [19] examined the impact of the layer thickness, printing angle, infill density, and nozzle temperature on the tensile and dynamic mechanical properties of PLA filament, highlighting the significant effect of the layer thickness on tensile strength.

Naveed [20] examined the tensile characteristics of FDM-printed parts at various linear raster angles (0°, 30°, 45°, 60°, and 90°), finding that a raster angle of 45° produced the highest average ultimate tensile strength (UTS), elongation at break, and elastic modulus. In another study, Naveed [21] analyzed two crisscross raster angles (0°/90° and 45°/45°) and infill speed using PLA and tough PLA materials. The crisscross raster angle of 45°/45° with a low infill speed achieved a superior average UTS and elongation at break for both materials, whereas the 45°/45° angle indicated higher material stiffness. Samykano [22] investigated the impact of the raster angle, layer thickness, and infill density on the tensile properties of FDM-printed PLA specimens. The study identified the infill density as the most critical factor influencing the UTS, while the elastic modulus, fracture strain, and toughness were primarily affected by both the infill density and layer thickness. The optimal tensile performance was achieved with an 80% infill density, a 40° raster angle, and a 0.3 mm layer thickness. Hikmat et al. [23] employed the signal to noise ratio (S/N) with the Taguchi L18 orthogonal array to identify optimal parameters and used analysis of variance (ANOVA) to analyze the significance. They found that significant factors included the build orientation, nozzle diameter, and infill density. The optimal configuration consisted of an on-edge orientation, 30° raster angle, 0.5 mm nozzle diameter, 200 °C extrusion temperature, 100% infill density, three shell layers, and 20 mm/s extrusion speed.

Lokesh et al. [24] investigated the influence of the layer thickness, raster angle, and build orientation on the mechanical properties of FDM-made PLA specimens using a Taguchi L9 orthogonal array. The study found that the layer thickness was the most significant factor, with a negative correlation to tensile strength. The highest UTS was achieved with a 0.1 mm layer height, a 30° raster angle, and a horizontal build orientation. Algarni [25] investigated how the raster angle and moisture content affect the mechanical properties of FDM-made PLA specimens. There were three raster angles (0°, 45°, and 90°) tested alongside varying moisture content percentages. The results indicate that specimens with a raster angle of 90° and a moisture content of 10% exhibit optimal strength and strain properties. Fontana et al. [26] investigated the impact of the layer thickness and infill rate on the tensile strength of FDM-printed PLA parts using a factorial design. ANOVA identified layer thickness as the dominant factor, and a regression model was developed to predict the tensile strength. Singh et al. [27] assessed how the infill percentage and raster pattern affect the tensile properties of PLA samples produced in different orientations: horizontal, on-edge, and vertical. ANOVA identified the build orientation and infill percentage as significant factors. The interaction between the build orientation and raster pattern also notably influenced the tensile strength. Samples produced in horizontal and on-edge orientations with a concentric pattern exhibited higher tensile strength than those printed vertically. Mani et al. [28] optimized the FDM parameters for PLA samples using a Taguchi L9 orthogonal array. They found that the optimum settings for maximum tensile strength were a layer thickness of 0.35 mm, infill density of 65%, and nozzle temperature of 220 °C. For hardness, the optimum parameters were a layer thickness of 0.25 mm, infill density of 65%, and nozzle temperature of 215 °C. The optimum parameters for surface finish were a layer thickness of 0.15 mm, infill density of 55%, and nozzle temperature of 210 °C.

In the study by Sahoo et al. [29], the Taguchi method was used to optimize the layer thickness, infill percentage, and print speed for tensile strength and hardness of FDM-produced PLA specimens. The layer thickness and infill percentage were the most influential factors. The optimal settings for tensile strength were 0.5 mm layer thickness, 35% infill, and 50 mm/s print speed, while for hardness, they were 0.1 mm layer thickness, 35% infill, and 60 mm/s print speed. In a study by Frunzaverde et al. [30], the influence of the layer thickness and filament color on the UTS and dimensional accuracy of FDM-made PLA specimens was examined. The black filament exhibited the highest UTS and better dimensional accuracy. It was also found that the UTS decreased as the layer thickness increased. Sandanamsamy et al. [31] studied the effect of nozzle temperature and raster angle on the mechanical properties of FDM-made PLA specimens. Higher raster angles and extrusion temperatures improved tensile strength, Young’s modulus, and yield strength. Maximum tensile strength was achieved with a 90° raster angle and 220 °C nozzle temperature. Ahmed et al. [32] used a Taguchi L18 orthogonal array to optimize the printing and post-processing parameters for FDM-made PLA specimens. The study found that the optimal conditions for UTS (37.15 MPa) included a 0.16 mm layer height, 90% infill density, gyroid infill pattern, 195 °C print temperature, and 90 °C annealing temperature.

When summarizing the literature review, it is abundantly clear that significant efforts have been made to explore the influence of process parameters on the tensile properties of FDM 3D-printed PLA specimens, with the ultimate goal of optimizing these parameters for enhanced tensile performance. However, notable inconsistencies persist in the findings regarding the impact of these parameters on tensile strength. For instance, some studies report that specimens printed with a flat build orientation exhibit the highest tensile strength, followed by on-edge and upright orientations [17,18], while others suggest that on-edge orientations result in superior tensile strength, with flat and upright orientations showing lower values [4,11,23,27]. Furthermore, the effect of layer thickness on tensile strength also varies across studies. Some research shows a positive correlation between thicker layers and enhanced tensile strength [16,28], whereas others observe a negative correlation, indicating that thicker layers led to reduced tensile strength [14,15,19,22,24,26,30]. Most studies that report varying trends in tensile strength employed regular PLA [4,11,14,15,16,17,18,22,23,24,27,28,30], with a few exploring PLA Tough [26] and PolyPlus PLA [19]. The filament diameter was predominantly reported as 1.75 mm [4,11,16,17,18,19,22,23,24,27,28], with a few studies specifying a tolerance of ±0.05 mm [14,15]. A 2.85 mm filament was reported in [30], while [26] did not report the diameter. The material density was commonly reported as 1.24 g/cm^3^ [4,11,16,23,24,27,28], while [15] reported 1.2 ± 0.02 g/cm^3^, although several studies omitted density data [14,17,18,19,22,26,30]. The presence of additives, such as pigments, was reported only in [30]. There is no compelling evidence to suggest that variations in PLA material properties account for the inconsistent tensile strength trends observed with build orientation and layer thickness. For example, the studies reported in [24,28], both using 1.75 mm filament and PLA with a density of 1.24 g/cm^3^, presented opposing trends regarding the layer thickness. Study [28] reported improved tensile strength with increased layer thickness, whereas study [24] found the opposite trend, with thinner layers yielding higher tensile strength. Furthermore, while color additives did not alter the general trend of decreasing tensile strength with increasing layer thickness, they influenced the degree of reduction. For instance, gray PLA showed a minimal reduction (4.77%), whereas black PLA exhibited a more significant decrease (23.41%) [30]. Another key aspect highlighted in the review is the influence of the raster angle on tensile strength. While several studies have extensively examined the effects of linear raster angles on tensile properties [13,15,20,22,25,31], there is limited investigation into the impact of alternative raster angles [21,23].

Therefore, further research is necessary to gain a more comprehensive understanding of how various process parameters influence the tensile properties of 3D-printed PLA components. It is important to identify the optimal combination of process parameter levels that can maximize tensile properties of PLA materials. In this study, the Taguchi method was utilized to systematically evaluate the effects of process parameters on the UTS and elastic modulus of PLA specimens produced through FDM technology. The primary objective was to identify the optimal process parameter levels that result in the highest UTS and elastic modulus. Specifically, this study focused on key parameters, including the layer thickness, build orientation, raster angle, and extrusion temperature. ANOVA was employed to assess the statistical significance of each parameter’s influence.

## 2. Materials and Methods

### 2.1. Printing Materials

In this investigation, PLA silver developed by filament PM (Plasty Mladeč) (Moravia, Czech Republic) was utilized [33]. PLA silver is well known for its ease of printing, suitability for large objects, eco-friendliness, and biodegradable properties. Its specifications are detailed in Table 1.

### 2.2. Design of Experiment (DOE)

Understanding how the process parameters of the FDM manufacturing method influence mechanical properties and optimizing them for desired outcomes is crucial for enhancing the performance and functionality of parts produced using this technology [34]. In this investigation, the Taguchi DOE using an orthogonal L9 array created with Minitab 2021 software was employed to analyze the influence of the process parameters and to determine the combination of levels of these parameters that achieve the optimum UTS and elastic modulus for PLA specimens produced through FDM technology. The process parameters examined, including the layer thickness, build orientation, raster angle, and extrusion temperature, along with their corresponding levels, are detailed in Table 2. A one-way ANOVA was conducted to examine the significance of these parameters in influencing the UTS and elastic modulus. The Taguchi method was selected for its structured and systematic approach, which minimizes the number of tests required, thereby conserving time and costs associated with the testing procedure [23]. Table 3 illustrates the parameters examined and the total number of experiments conducted using the orthogonal $L9\;(3^{4})$ array. To ensure the reproducibility and reliability of the measurements, each experimental run consisted of 3 specimens, resulting in a total of 27 specimens for the nine experimental runs. This replication was implemented to minimize random errors and validate the consistency of the results. The S/N ratio is a statistical measure used in Taguchi optimization to evaluate the effectiveness of process parameters in achieving the desired response or performance while minimizing the effects of noise. In this study, all desired responses were quality attributes of the ‘larger is better’ type, and Equation (1) was utilized to estimate the S/N ratios for these attributes.

(1) $$S/N=-10\log\left(\frac{1}{n}\sum_{i=1}^{n}\frac{1}{y_{i}^{2}}\right)$$

where S/N is the signal-to-noise ratio; y is the output response value; i is 1, 2, 3, . . . n, where, in this study, n=3 because each of the nine experimental runs included three duplicate tests.

### 2.3. Specimen Printing

**Figure 1:**
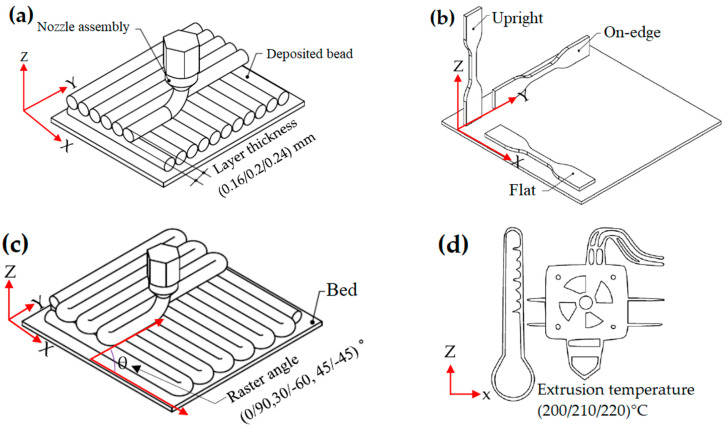
(**a**) The layer thickness, (**b**) build orientation for tensile test, (**c**) raster angle, and (**d**) extrusion temperature.

The tensile specimens were fabricated using a Printo H3 3D printing system (Printo3D, Lusowo, Poland). The CAD model of the test specimen was designed using SolidWorks 2021 software, adhering to the dimensions specified in the ISO 527-2 Type 5A standard test method [35], and subsequently converted to STL format. The STL file was sliced using Ultimaker Cura 4.8 software, which generated the G-code (instructions for the 3D printing system) based on a specific set of printing conditions. The selected FDM process parameters, with their respective levels, are visualized in Figure 1a–d, including three distinct levels for each parameter: layer thicknesses of 0.16, 0.2, and 0.24 mm; build orientations of flat, on-edge, and upright; raster angles of 0°/90°, 30°/−60°, and 45°/−45°; and extrusion temperatures of 200 °C, 210 °C, and 220 °C. The extrusion temperatures were chosen based on the PLA filament supplier’s recommended range of 200 °C to 220 °C to avoid potential issues such as improper bonding or inconsistent extrusion, which can arise from temperatures that are too low or too high [33,36]. The layer thicknesses of 0.16 mm and 0.2 mm were selected based on previous studies [32], while the 0.24 mm thickness was included to investigate the influence of higher layer thicknesses on tensile properties [37]. Similarly, the raster angles and build orientations were selected based on previous research [4,23]. The common printing parameters, listed in Table 4, were kept constant while 3D printing all specimens using an FDM printer.

### 2.4. Tensile Test

**Figure 2:**
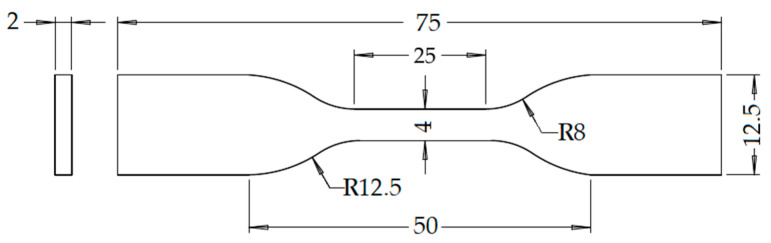
Tensile specimen of Type 5A according to ISO 527-2 standards, with dimensions in mm [35].

**Figure 3:**
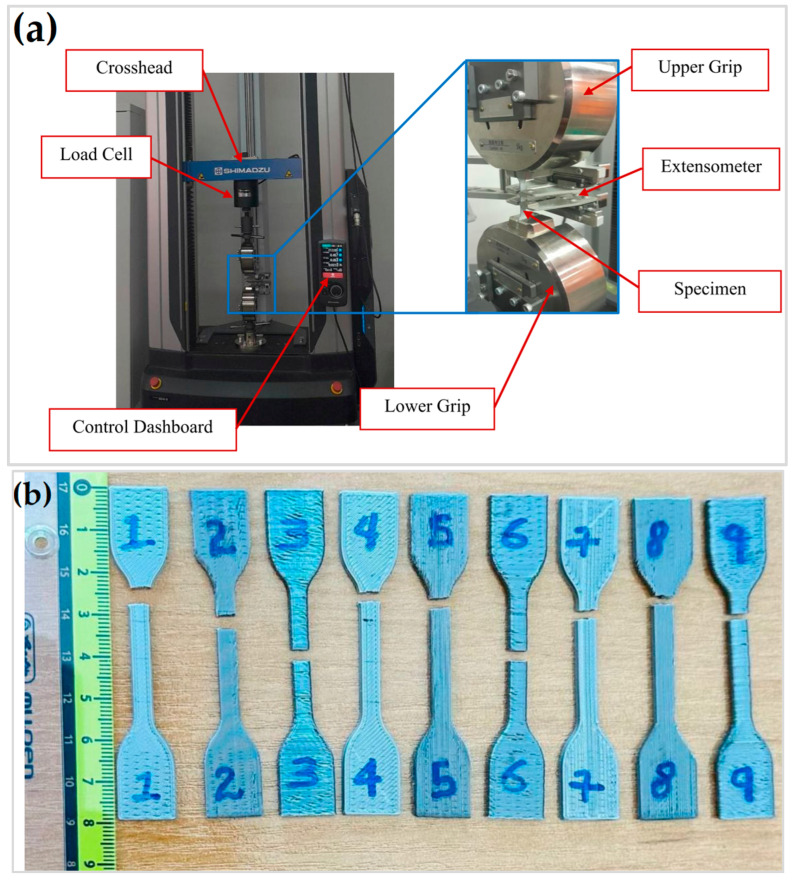
(**a**) Tensile testing setup and (**b**) tested specimens.

The tensile test was performed using a Shimadzu universal testing machine (Shimadzu Autograph AGSX, Shimadzu Corporation, Kyoto, Japan) equipped with a 10 kN load cell, following the ISO 527-2 Type 5A standard [35]. Figure 2 shows the dimensions for the ISO 527-2 Type 5A standard, while Figure 3a,b indicates the tensile testing setup and the tested specimens, respectively. Throughout the test, the crosshead speed was maintained at a constant 1 mm/min. The UTS represents the maximum load per unit area a material can withstand under tensile stress before failure occurs. Determining the UTS involves analyzing the stress–strain curve obtained from the tensile test to identify the peak point. Additionally, the stress–strain curve provides insights into other properties, such as the elastic modulus, which signifies the material’s resistance to elastic deformation when subjected to force. The elastic modulus is calculated as the slope of the stress–strain curve within the elastic deformation range, typically between 0.05% and 0.25% strain. A steeply rising curve indicates a high elastic modulus, whereas a gently rising curve indicates a lower elastic modulus [22].

### 2.5. Fracture Surface Analysis via Scanning Electron Microscopy

A scanning electron microscope (FE-SEM, Gemini, Leo 1525, Zeiss, Thornwood, NY, USA) operating at 15 kV was used to examine the fracture surfaces of PLA specimens printed within experimental Runs 1 and 6. Before imaging, the specimens were carefully prepared by cutting them to precise dimensions and coating them with platinum.

## 3. Results and Discussion

### 3.1. Experimental Results

**Figure 4:**
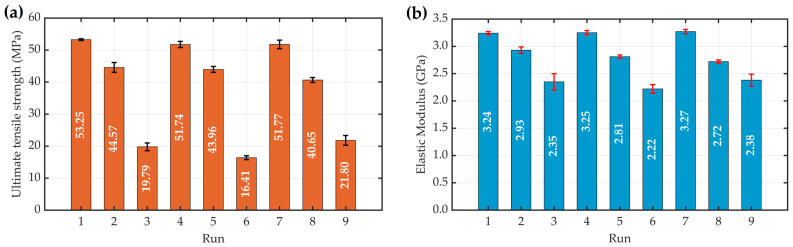
Graphical representation of experimental results: (**a**) average UTS and (**b**) average elastic modulus, with error bars representing the standard deviations based on three specimens per experimental run.

The UTS and elastic modulus results from tensile tests on three specimens for each of the nine experimental runs in the Taguchi L9 orthogonal array, along with their average values (±standard deviations), are detailed in Table A1 (Appendix A). These results are graphically represented in Figure 4a,b, where the average UTS and average elastic modulus are shown with error bars representing the standard deviations, respectively. The highest average UTS measured was 53.25 MPa obtained with Run 1, while the lowest was 16.41 MPa obtained with Run 6 (Figure 4a). For the elastic modulus, the highest average value measured was 3.27 GPa, obtained with Run 7, and the lowest was 2.22 GPa, obtained with Run 6 (Figure 4b). The range of values observed for both the UTS and elastic modulus highlights the variability in performance among specimens manufactured with different sets of process parameters of the FDM 3D printing system. Notably, specimens with the highest average UTS and elastic modulus were fabricated with a flat build orientation, while those with an upright build orientation displayed the lowest values for both responses, indicating the significant influence of build orientation on material performance. These results are in agreement with prior studies by N. H. Patadiya et al. [17] and Domenico Corapi et al. [18], which investigated the tensile properties of PLA specimens and found that specimens printed with a flat orientation exhibited the highest tensile strength.

**Figure 5:**
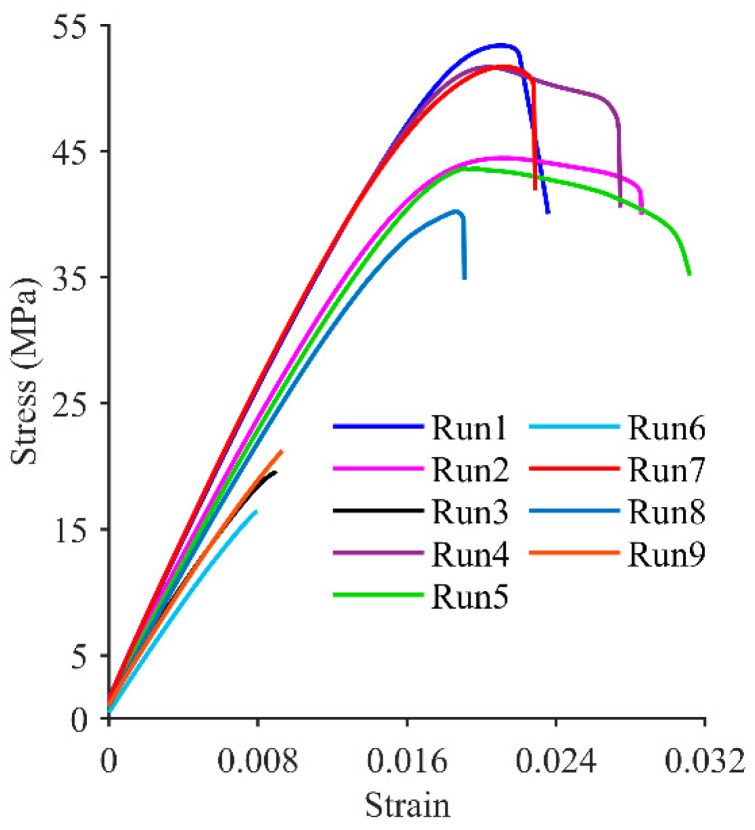
Average stress–strain curves.

The stress–strain curves shown in Figure 5 indicate that the average tensile behavior of the samples exhibits significant anisotropy, with build orientation playing a dominant role in influencing the material response compared to layer thickness and raster angle. Specimens printed with an upright build orientation (Runs 3, 6, and 9) displayed brittle behavior, characterized by a linear elastic region up to stresses of 19.5 MPa (Run 3), 16.3 MPa (Run 6), and 21.7 MPa (Run 9), followed by abrupt failure with minimal plastic deformation. In contrast, specimens printed with a flat build orientation (Runs 1, 4, and 7) exhibited ductile behavior, showing a more extensive linear elastic region up to stresses of 49.6 MPa (Run 1), 43.7 MPa (Run 4), and 46.2 MPa (Run 7), followed by significant plastic deformation before failure. After the UTS, a reduction in stress was observed until failure occurred. Specimens printed with an on-edge build orientation (Runs 2, 5, and 8) also demonstrated ductile behavior, exhibiting a linear elastic region up to stresses of 36.5 MPa (Run 2), 34.2 MPa (Run 5), and 35.2 MPa (Run 8), followed by moderate plastic deformation before failure. Similarly, after reaching the UTS, a reduction in stress was observed until failure occurred. These findings align with the previous studies by Caminero et al. [38], Chacón et al. [4], and Rybachuk et al. [39]. Caminero et al. demonstrated that build orientation is a critical factor influencing the elasticity and plasticity of 3D-printed materials. PLA printed in flat and on-edge orientations exhibited more ductile behavior and enhanced tensile strength, whereas upright prints exhibited rapid failure with minimal plastic deformation, characteristic of brittle fracture [38]. Similarly, Chacón et al. reported that the stress–strain behavior of PLA is significantly influenced by build orientation, with upright samples failing suddenly and with minimal plastic deformation, while flat and on-edge orientations exhibited superior mechanical properties and increased ductility [4]. Rybachuk et al.’s findings on ABS further support this conclusion, noting that upright samples experienced sudden failure with limited plastic deformation, while flat orientations displayed improved ductility and higher tensile strength [39].

**Figure 6:**
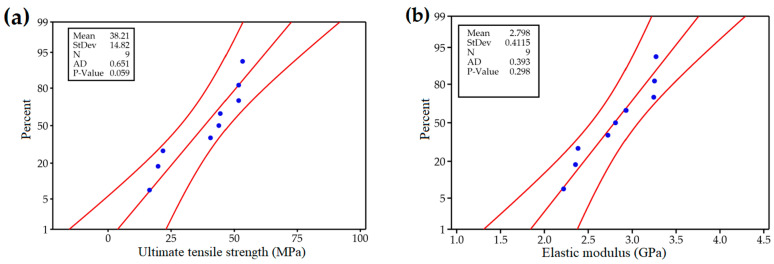
Normal probability plot of (**a**) ultimate tensile strength and (**b**) elastic modulus.

Figure 6a,b depicts the UTS and elastic modulus values of tensile specimens produced using controlled FDM 3D printing process parameters, represented as percentages of the normal distribution, respectively. In Figure 6, the blue points represent the observed data, the outer red lines denote the 95% confidence interval limits, and the red center line represents the fitted line of the normal distribution. The plots help to determine whether the data conform to a normal distribution by assessing the alignment of data points with the centerline. To further verify normality, the Anderson–Darling (AD) test was applied, where the null hypothesis of normality is accepted if the *p*-value exceeds 0.05 [40]. In this case, the *p*-value for UTS is 0.059, and that for the elastic modulus is 0.298. Both *p*-values are above 0.05, which confirms that there is no significant evidence against the hypothesis that the data are normally distributed. The normal probability plots (Figure 6a,b) show that all data points align closely with the fitted line, indicating no outliers. This indicates that the residuals are evenly distributed in all runs, which confirms that the data follow a normal distribution, and the hypothesis is accepted. This suggests that the residuals are evenly distributed across all runs, confirming that the data follow a normal distribution and supporting the use of ANOVA and regression models for further analysis.

### 3.2. Taguchi Optimization by S/N Ratios

The Taguchi method is a statistical technique that requires fewer experimental runs, making it both cost-effective and time efficient. It employs three essential tools: orthogonal experimental design for efficient experimentation, S/N ratios to measure performance, and ANOVA to identify significant factors. This approach helps optimize the settings, improve quality, and reduce variability, thereby enhancing process robustness and consistency [23,24,41]. The influence of the FDM process parameters (layer thickness, build orientation, raster angle, and extrusion temperature) on the UTS and elastic modulus was investigated by analyzing the S/N ratio for each experimental run within the orthogonal L9 Taguchi array, as presented in Table 5. In this study, the S/N ratio, computed using Minitab software, adhered to the ‘larger is better’ quality attribute paradigm to ascertain the optimum levels for each process parameter aimed at maximizing the UTS and elastic modulus.

**Figure 7:**
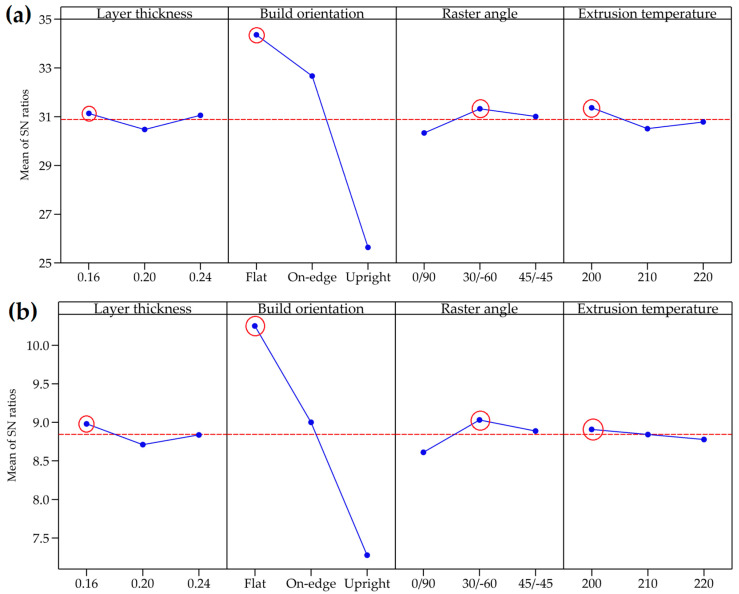
Main effect plot for the signal-to-noise ratio (larger is better) of (**a**) ultimate tensile strength and (**b**) elastic modulus.

The levels of process parameters that give the optimum response are determined by calculating the average S/N ratios for each response, with the maximum S/N ratios indicating the levels that result in the optimum response, according to the ‘larger is better’ quality attribute. Research by Hikmat et al. [23] and Lokesh et al. [24] utilizes the S/N ratio to explore various process parameters in FDM 3D printing of PLA materials. Figure 7a,b depicts the main effect plots for the S/N ratios of the UTS and elastic modulus, respectively. The red dotted line in Figure 7a,b represents the reference line. Process parameter levels above this line correspond to higher S/N ratios, indicating that these levels are likely to be sufficient for achieving high UTS and elastic modulus. In contrast, levels below the reference line are associated with lower S/N ratios and may be inadequate for achieving the desired material properties. The influence of controlled process parameters on the UTS and elastic modulus performance, as measured by the S/N ratios, is presented in Table 6. The delta range (d∆) between the highest and lowest values in this table quantifies the influence of each process parameter. The significance of the parameters is ranked based on the magnitude of the delta values from highest to lowest. Build orientation exhibits the most significant influence on UTS, with a ∆S/N ratio of 8.72 dβ, followed by raster angle and extrusion temperature, with ∆S/N ratios of 0.99 dβ and 0.86 dβ, respectively. Layer thickness shows the least influence, marked by a ∆S/N of 0.66 dβ. For the elastic modulus, build orientation has the most substantial influence, with a ∆S/N of 2.973 dβ, followed by raster angle and layer thickness, which have ∆S/N values of 0.420 dβ and 0.270 dβ, respectively. Extrusion temperature exerts the least influence on elastic modulus, with a ∆S/N of 0.130 dβ. As depicted in Figure 7a,b and Table 6, the combination of levels achieving optimum values for both the UTS and elastic modulus is L1B1R2T1, meaning layer thickness at level 1 (0.16 mm), build orientation at level 1 (flat), raster angle at level 2 (30°/−60°), and extrusion temperature at level 1 (200 °C).

**Figure 8:**
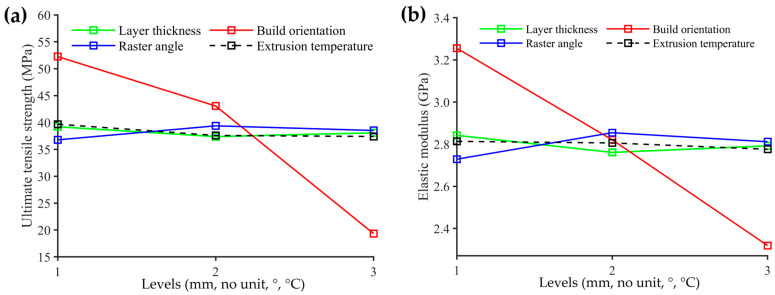
Main effect plot for mean of (**a**) ultimate tensile strength and (**b**) elastic modulus.

Figure 8a,b shows the mean values of the UTS and elastic modulus across different levels of controlled process parameters, including the layer thickness, build orientation, raster angle, and extrusion temperature, respectively. These diagrams are used to compare the relative response and to analyze the influence of the individual process parameters on the UTS and elastic modulus. The plot for the mean value of UTS at three levels of layer thickness revealed an initial decrease followed by an increase, as represented by the green line in Figure 8a. The highest mean value of UTS observed was 39.2 MPa at level 1 (0.16 mm), and it decreased to 37.37 MPa at level 2 (0.20 mm), followed by a slight increase to 38.07 MPa at level 3 (0.24 mm). This finding was consistent with those of previous studies by Lanzotti et al. [42]. Thinner layers enhance UTS because the shorter distance between the nozzle and the previously deposited layer allows for more efficient heating and stronger fusion between sequential layers, resulting in maximum strength [19]. In contrast, thicker layers increase the distance between the nozzle and the deposited layer, causing uneven heating, reduced pressure, and a higher likelihood of voids, which weakens the tensile strength [19,24]. However, as Chacon et al. and Bardiya noted, increasing the layer thickness reduces the number of layers required to fabricate a part, minimizing deformations from stress accumulation due to fewer heating and cooling cycles during printing, which in turn enhances the tensile strength [4,43].

The red line in Figure 8a shows the mean value of UTS versus the levels of the building orientation. It was found that specimens printed with a flat orientation (level 1) exhibited the highest mean value of UTS (52.25 MPa), which was much higher than the mean value of UTS (43.06 MPa) for specimens printed with an on-edge orientation (level 2), showing a difference of 21.56%. Similarly, it was higher than the mean value (19.33 MPa) of specimens printed with an upright orientation (level 3), with a difference of 170.46%. The results of this study were consistent with those of previous research [17,18,44], which emphasized the influence of build orientation on the tensile strength of parts produced by FDM 3D printing. The parts exhibit higher strength when the extruded rods are oriented parallel to the direction of the applied load, which optimizes the transfer of stress to the material and improves its resistance under loading. In flat and on-edge build orientations, many long rods extrude in the direction of the same applied load during tensile testing, thereby requiring greater force to break the specimens [44]. Moreover, flat orientation-printed specimens provide a broad interface area between the layers, enhancing their ability to withstand tensile loads compared to those printed with an on-edge build orientation [45]. For upright orientation-printed specimens, the load direction is perpendicular to the direction of the extruded rods, indicating that the bonds between the rods are weaker and that inter-layer fractures occur easily during tensile testing [4,23,44].

The blue line in Figure 8a illustrates the variability in the mean value of UTS across the alternative raster angles studied. Specimens printed with a 30°/−60° raster angle (level 1) exhibited the highest mean value of UTS (39.37 MPa), showing a 6.92% increase compared to the mean value of UTS (38.51 MPa) for specimens printed with a 45°/−45° raster angle (level 2). Additionally, it demonstrated a 7.07% increase compared to the mean value of UTS (36.77 MPa) for specimens printed with a 0°/90° raster angle (level 3). This study showed that the 30°/−60° raster angle provides superior performance among the tested raster orientations because of its deposited rod configuration. Specimens printed with a 30°/−60° raster angle have a greater number of parallel deposited rods compared to the 0°/90° raster angle and feature longer parallel deposited rods than the 45°/−45° raster angle. These rods are aligned to the tensile loading direction, enhancing the resistance of the specimens to breaking during testing. This finding is in agreement with Hikmat et al. [23] and Hasanzadeh et al. [46].

As shown in Figure 8a, the black dashed line represents the mean value of UTS versus the levels of extrusion temperature, which decreased as the extrusion temperature increased from 200 °C (level 1) to 220 °C (level 3). Specifically, at an extrusion temperature of 200 °C, the mean UTS was 39.67 MPa, whereas at 220 °C, it was 37.39 MPa, representing a 5.75% decrease in the mean value of UTS. This result is consistent with the findings of Ahmed et al. [32] and Shergill et al. [47], which indicate a negative correlation between extrusion temperature and UTS. The extrusion temperature is crucial for achieving optimal fusion strength between the layers in 3D printing. The appropriate temperature ensures that the extruded material remains molten enough to flow seamlessly and bond with the previously deposited layer, resulting in a robust and cohesive structure. Optimal fusion is achieved within a specific temperature range, known as the temperature threshold, where the material’s flow properties enhance the UTS. However, elevated temperatures can cause degradation of the polymer chains and increased material shrinkage due to higher thermal expansion before cooling, ultimately leading to a reduced UTS [48].

As indicated in Figure 8b, the mean value of the elastic modulus varied significantly with different process parameter levels. The layer thickness had a notable impact, as indicated by the green line. The 0.16 mm thickness (level 1) yielded the highest mean value of 2.84 GPa, a 1.78% increase compared to the 0.24 mm thickness (level 3), which measured 2.79 GPa. Thinner layers lead to higher elastic modulus values, suggesting finer layer deposition enhances material stiffness due to better bonding and reduced voids between layers [49]. The red line represents the mean value of the elastic modulus versus build orientation, indicating that the build orientation had the most significant influence. The flat orientation (level 1) achieved the highest mean value of 3.26 GPa, 40.35% higher than the upright orientation (level 3) measuring 2.32 GPa, and 15.41% higher than the on-edge orientation (level 2) measuring 2.82 GPa. When the layers are laid flat, the bonding between them is optimized, enhancing mechanical performance. Conversely, the upright orientation results in weaker interlayer bonding and thus a lower elastic modulus [50]. The raster angle also played a pivotal role, as indicated by the blue line. The 30°/−60° angle (level 2) produced the highest mean value of 2.85 GPa, a 4.61% increase over the 0°/90° angle (level 1) measuring 2.73 GPa, and a 1.53% increase over the 45°/−45° angle (level 3) measuring 2.81 GPa. The 30°/−60° raster angle configuration optimizes the load distribution and enhances overall material integrity. The extrusion temperature affected the elastic modulus, as shown by the black dashed line. The extrusion temperature of 200 °C (level 1) showed the highest mean value of 2.81 GPa, a 1.35% increase over 220 °C (level 3) measuring 2.78 GPa, and a slight increase over 210 °C (level 2) measuring 2.81 GPa. While the temperature differences are relatively small, maintaining a lower extrusion temperature can enhance material properties [48].

### 3.3. ANOVA and Interaction Plots

To analyze the primary impact of the process parameters on the UTS and elastic modulus, a one-way ANOVA was conducted with a 95% confidence level. The analysis generated a *p*-value for each parameter to determine its significance. A *p*-value of less than 0.05 signifies statistical significance, indicating that the parameter has a statistically significant influence. In contrast, a *p*-value greater than 0.05 means the parameter impact is not statistically significant [23]. The ANOVA results in Table 7 show that layer thickness, build orientation, raster angle, and extrusion temperature were statistically significant in influencing UTS, with *p*-values below 0.05. Among these, build orientation was the most significant, contributing 98.16% to the observed variation in UTS, followed by raster angle (0.6%), extrusion temperature (0.54%), and layer thickness (0.29%). For the elastic modulus, both the build orientation and raster angle were statistically significant, contributing 94.83% and 1.76% to the observed variation, respectively. Layer thickness and extrusion temperature were statistically insignificant and made minor contributions. These findings underscore the critical role of the build orientation and raster angle in influencing the mechanical properties of FDM-fabricated PLA parts.

**Figure 9:**
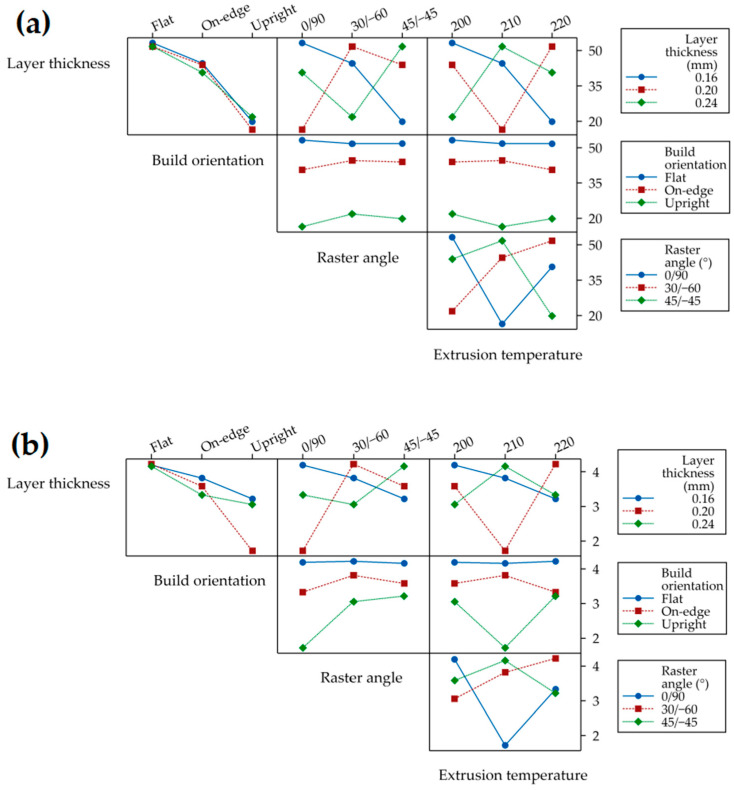
Interaction plot of (**a**) ultimate tensile strength (MPa) and (**b**) elastic modulus (GPa).

Interaction plots are valuable tools for visualizing how the combined effects of two or more variables influence the response values, often under controlled conditions. These plots were used to assess whether the effect of one variable on the response changed depending on the level of the other variable. They reveal relationships by displaying two types of line: parallel lines indicate no interaction between variables, whereas nonparallel lines suggest significant interactions [51]. Figure 9a,b depicts the interaction plots for the UTS and elastic modulus, respectively, with respect to process parameters such as the layer thickness, build orientation, raster angle, and extrusion temperature. Notably, none of the lines in these plots were parallel when these factors were combined, indicating strong interactions that significantly influenced both the UTS and elastic modulus. The highest values of UTS and elastic modulus were typically achieved with a flat build orientation, layer thickness of 0.16 mm, raster angle of 0°/90°, and extrusion temperature of 200 °C.

### 3.4. Linear Regression, Confirmation, and Contour Plot

To obtain the regression equation and coefficient of determination for the UTS and elastic modulus, a 95% confidence level was utilized. The regression equations for UTS and elastic modulus were formulated with raster angle and build orientation as categorical variables and layer thickness and extrusion temperature as continuous variables. Ahmed et al. [32] developed the regression model by classifying the 3D printing parameters into categorical and continuous. For Equations (2) and (3), the R-squared (R-sq) coefficients indicate that the model accurately predicted the UTS (R-sq = 99.71%) and the elastic modulus R-sq = 99.52%).

(2) $$UTS\left(\mathrm{MPa}\right)=A-14.2\times L-0.1139\times T$$

(3) Elastic modulus (GPa)=B−0.621×L−0.00188×T

where L is the layer thickness in mm and T is the extrusion temperature in degrees Celsius. The values of A and B vary for different combinations of raster angle and build orientation, as shown in Equation (4).

(4) $$\;A=\left\{\begin{array}{c} 77.5,\;for\;Flat\;(0{}^{\circ}/90{}^{\circ})\\ 80.2,\;for\;Flat\;(30{}^{\circ}/-60{}^{\circ})\\ 79.3,\;for\;Flat\;(45{}^{\circ}/-45{}^{\circ})\\ 68.4,\;for\;On-edge\;(0{}^{\circ}/90{}^{\circ})\\ 71.0,\;for\;On-edge\;(30{}^{\circ}/-60{}^{\circ})\\ 70.1,\;for\;On-edge\;(45{}^{\circ}/-45{}^{\circ})\\ 44.6,\;for\;Upright\;(0{}^{\circ}/90{}^{\circ})\\ 47.2,\;for\;Upright\;(30{}^{\circ}/-60{}^{\circ})\\ 46.4,\;for\;Upright\;(45{}^{\circ}/-45{}^{\circ}) \end{array}\right.\mathrm{and}\;B=\left\{\begin{array}{c} 3.705,\;for\;Flat\;(0{}^{\circ}/90{}^{\circ})\\ 3.831,\;for\;Flat\;(30{}^{\circ}/-60{}^{\circ})\\ 3.788,\;for\;Flat\;(45{}^{\circ}/-45{}^{\circ})\\ 3.271,\;for\;On-edge\;(0{}^{\circ}/90{}^{\circ})\\ 3.396,\;for\;On-edge\;(30{}^{\circ}/-60{}^{\circ})\\ 3.353,\;for\;On-edge\;(45{}^{\circ}/-45{}^{\circ})\\ 2.769,\;for\;Upright\;(0{}^{\circ}/90{}^{\circ})\\ 2.894,\;for\;Upright\;(30{}^{\circ}/-60{}^{\circ})\\ 2.851,\;for\;Upright\;(45{}^{\circ}/-45{}^{\circ}) \end{array}\right.$$

**Figure 10:**
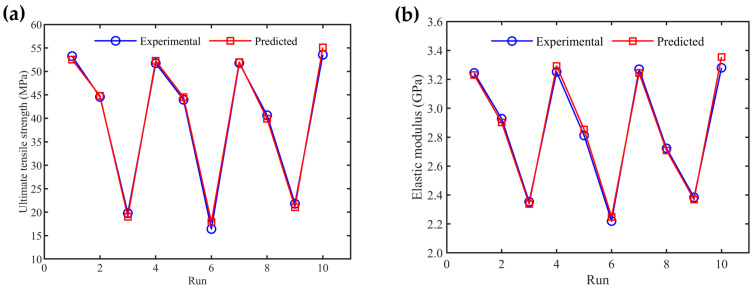
Comparison of predicted values with experimental results for (**a**) ultimate tensile strength and (**b**) elastic modulus.

Figure 10a,b shows a comparative analysis of experimental and predicted values for the UTS and elastic modulus, respectively. The graphs illustrate the results from nine experimental runs alongside their corresponding regression model predictions, as well as experimental Run 10, which pertains to a specimen printed with optimal process parameters (layer thickness = 0.16 mm, raster angle = 30°/−60°, extrusion temperature = 200 °C) and its prediction. The mean absolute percentage errors for all nine experimental runs are minimal: 2.54% for UTS and 0.89% for elastic modulus. These low error values demonstrate that the proposed regression models accurately and effectively predict the UTS and elastic modulus for PLA materials.

**Figure 11:**
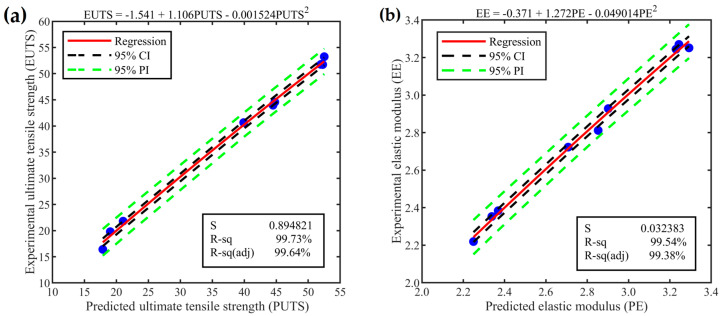
The plot depicts the fitted quadratic regression line, illustrating the relationship between predicted and experimental values for (**a**) ultimate tensile strength and (**b**) elastic modulus.

The relationship between the predicted values and experimental results was investigated via quadratic regression analysis, as shown in Figure 11a,b for the UTS and elastic modulus, respectively. As the experimental results are closer to the regression curve, the R-Sq values increase, which indicates the level of confidence in the prediction models. In Figure 11, blue dots denote the experimental results, green dashed lines delineate the prediction intervals (PIs), black dashed lines indicate the confidence intervals (CIs), and the red solid line represents the regression curve. When comparing the experimental outcomes with the predicted values for UTS and elastic modulus, the confidence interval limits for the predicted values are 99.73% and 99.54%, respectively. The predicted values for UTS and elastic modulus were within the 95% confidence interval limits and were deemed acceptable when compared to the experimental results.

The combination of process parameter levels that yield the optimum UTS and elastic modulus, as determined by the Taguchi L9 orthogonal array, was not found in the experiments performed. For validation purposes, experiments were conducted to evaluate the effectiveness of both the Taguchi method and the linear regression model using a specimen printed with L1B1R2T1 (layer thickness = 0.16 mm, raster angle = 30°/−60°, and extrusion temperature = 200 °C) for both UTS and elastic modulus. The observed differences between predicted values by the Taguchi method and experimentally obtained values, as well as between predicted values by the linear regression model and experimentally obtained values, are presented in Table 8. The percentage error for the Taguchi method is 4.33% for UTS and 2.74% for elastic modulus, while the percentage error for the linear regression model is 2.95% for UTS and 2.44% for elastic modulus.

**Figure 12:**
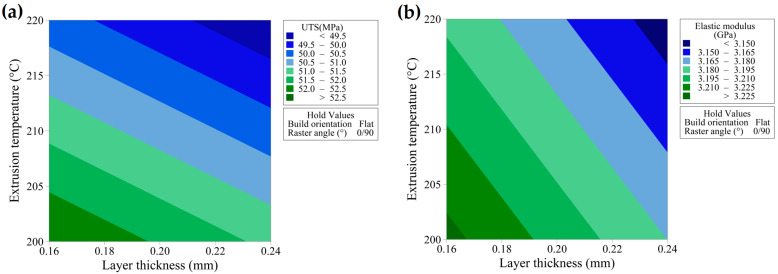
Contour plot showing continuous parameters (layer thickness and extrusion temperature) versus (**a**) ultimate tensile strength and (**b**) elastic modulus.

Contour plots are a valuable means of visualizing the relationship between a response variable and two continuous parameters [40]. In models that contain both continuous and categorical variables, the categorical variables are kept constant while illustrating the influence of the continuous factors on the response. These plots effectively show how variations in the process parameters, shown on the X and Y axes, affect the response, with shaded contours indicating these relationships. Figure 12a,b shows contour plots depicting the UTS and elastic modulus values for different levels of layer thickness and extrusion temperatures at constant build orientation and raster angle. As can be observed, both the UTS and the elastic modulus increase as the layer thickness and extrusion temperature decrease. The highest values for UTS and elastic modulus were observed at a layer thickness of 0.16 mm and an extrusion temperature of 200 °C.

### 3.5. SEM Examination of Fractured Surfaces

The Taguchi analysis identified build orientation as the most influential factor affecting the UTS of FDM-printed specimens. To further explore these effects, SEM was utilized to examine the fractured surfaces of specimens with the highest and lowest UTS. Specimens from experimental Run 1, fabricated with a 0°/90° raster angle, 0.16 mm layer thickness, 200 °C extrusion temperature, and flat build orientation, showed the highest UTS of 53.25 MPa. In contrast, specimens from experimental Run 6, printed with a 0°/90° raster angle, 0.2 mm layer thickness, 210 °C extrusion temperature, and upright build orientation, recorded the lowest UTS of 16.41 MPa.

**Figure 13:**
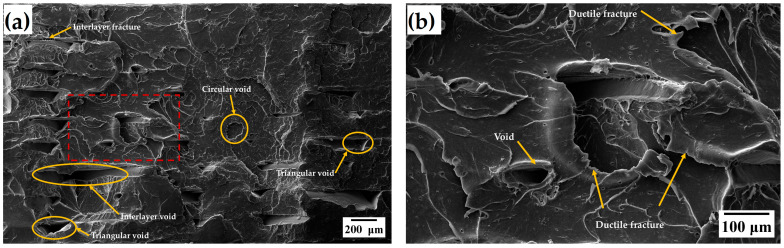
SEM images of fractured surfaces of specimens from experimental Run 1, where (**a**) represents the surface at 100× magnification, and (**b**) shows an enlarged view of the selected region (highlighted by the red dotted rectangle) at 300× magnification.

The SEM overview of the specimens in Figure 13a reveals various void types, including circular voids, interlayer voids, interlayer fractures, and triangular voids. These triangular voids are mainly characterized by their elongated shapes, originating from voids between adjacent bead rods and extending along the layer boundaries. These triangular voids serve as key points where failure begins, leading to the formation of horse-hoof-shaped voids. The high-magnification SEM image (Figure 13b) demonstrates how these voids significantly widen under tensile loading, both in the interlayer and in-layer regions. This widening, along with necking and fibril formation near the void openings, signifies a ductile fracture mechanism.

**Figure 14:**
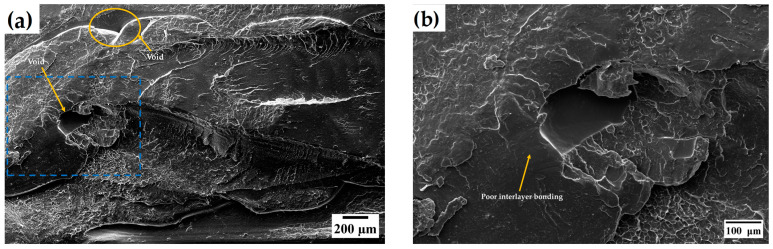
SEM images of fractured surfaces of specimens from experimental Run 6, where (**a**) represents the surface at 100× magnification, and (**b**) shows an enlarged view of the selected region (highlighted by the blue dotted rectangle) at 300× magnification.

On the other hand, Figure 14a illustrates a fracture surface from experimental Run 6 that appears smooth, with no distinct separation between layers, implying better integration. However, crater-like voids can be observed (Figure 14b), which occurred due to air gaps between layers, contributing to brittle fracture. The fracture surface is almost perpendicular to the applied load, which causes a failure in the interlayer fusion bonds, indicating predominantly brittle behavior.

Even though specimens from experimental Run 1 exhibited more voids on the fracture surface than those from Run 6, they displayed a ductile fracture pattern and achieved the highest UTS due to the alignment of the raster rods with the tensile force. In contrast, specimens from experimental Run 6 suffered from weak interlayer bonding and brittle failure because the raster rods were oriented perpendicular to the tensile load.

## 4. Conclusions

This study systematically investigated the influence of key process parameters in FDM technology, specifically the layer thickness, build orientation, raster angle, and print extrusion temperature, on the mechanical properties of PLA specimens, with a focus on the UTS and elastic modulus. Using a rigorous experimental and statistical approach that included the Taguchi method, ANOVA, and regression analyses, several significant findings emerged. UTS exhibited notable variations, ranging from 16.41 MPa to 53.25 MPa, while the elastic modulus varied from 2.22 GPa to 3.27 GPa. Normal probability plots showed that all data points closely aligned along a straight line and were uniformly distributed, indicating the absence of outliers.

Taguchi method analysis revealed that build orientation was the most significant factor affecting both the UTS and elastic modulus, with the flat orientation yielding the best performance for both properties. For UTS, ANOVA showed that build orientation, raster angle, and extrusion temperature significantly influenced UTS, with build orientation contributing 98.16% of the variation. For elastic modulus, build orientation and raster angle were the most significant factors, contributing 94.83% and 1.76%, respectively.

The optimal process parameters, determined through S/N ratio analysis, were a 0.16 mm layer thickness, flat build orientation, 30°/−60° raster angle, and 200 °C extrusion temperature. These parameters yielded UTS values of 55.11 MPa (from the regression equation) and 55.85 MPa (from the Taguchi method), with error percentages of 2.95% and 4.33%, respectively, compared to the experimental value of 53.53 MPa. Similarly, the predicted elastic modulus values showed an error percentage of 2.74% when compared to experimental results, demonstrating the model’s predictive accuracy. The regression models demonstrated strong predictive capability, with high coefficients of determination (R-sq = 99.71% for UTS and R-sq = 99.52% for elastic modulus). SEM images further highlighted that variations in the process parameters significantly impact filament adhesion and fracture patterns, underscoring the importance of precise parameter control in FDM printing for enhanced structural performance. Future research should explore a broader range of process parameters, material properties, and additives, incorporating fractional descriptions of temperature effects to better understand their influence on the mechanical performance of 3D-printed parts, such as fracture behavior, bending, and hardness.

## Figures and Tables

**Table 1:** Detailed information about PLA silver filaments.

Property	Value
Color	Silver
Diameter (mm)	1.7 ± 0.005
$\mathrm{Density}\;\left(g/{\mathrm{cm}}^{3}\right)$	1.24
Melt Flow Index (g/10 min)	6
Heat Bending Temperature (°C)	20–60
Printing Temperature (°C)	200–220
Tensile Modulus (GPa)	3.41
Elongation at Break (%)	2.3
Tensile Strength (MPa)	59.4

**Table 2:** Controlled process parameters and their ranges.

Name of Parameters		Levels		
		1	2	3
L	Layer thickness (mm)	0.16	0.2	0.24
B	Build orientation	Flat	On-edge	Upright
R	Raster angle (°)	0/90	30/−60	45/−45
T	Extrusion temperature (°C)	200	210	220

**Table 3:** Taguchi L9 ($3^{4}$) orthogonal array.

Run	Layer Thickness (mm)	Build Orientation	Raster Angle (°)	Extrusion Temperature (°C)
1	0.16	Flat	0/90	200
2	0.16	On-edge	30/−60	210
3	0.16	Upright	45/−45	220
4	0.2	Flat	30/−60	220
5	0.2	On-edge	45/−45	200
6	0.2	Upright	0/90	210
7	0.24	Flat	45/−45	210
8	0.24	On-edge	0/90	220
9	0.24	Upright	30/−60	200

**Table 4:** FDM printer and common 3D printing parameters.

Name of Parameter	Value
Model	Printo H3
Working area (mm)	200 × 200 × 205
Nozzle diameter (mm)	0.4
Bed temperature (°C)	60
Printing speed (mm/s)	50
Infill pattern	lines
Infill percentage (%)	100
Wall thickness (mm)	0.24
Fan speed (%)	100

**Table 5:** S/N ratios for ultimate tensile strength and elastic modulus.

Run	UTS (MPa)	S/N Ratio (dβ)	Elastic Modulus (GPa)	S/N Ratio (dβ)
1	53.25	34.53	3.24	10.22
2	44.57	32.97	2.93	9.33
3	19.79	25.90	2.35	7.39
4	51.74	34.27	3.25	10.24
5	43.96	32.86	2.81	8.98
6	16.41	24.29	2.22	6.91
7	51.77	34.28	3.27	10.29
8	40.65	32.18	2.72	8.70

**Table 6:** Average performance of S/N ratio (larger is better).

Response	Level	L→Layer Thickness (mm)	B→Build Orientation	R→Raster Angle (°)	T→Extrusion Temperature (°C)
UTS (MPa)	1	31.13	34.36	30.33	31.37
	2	30.47	32.67	31.32	30.51
	3	31.06	25.64	31.01	30.78
	Delta	0.66	8.72	0.99	0.86
	Rank	4	1	2	3
Elastic modulus (GPa)	1	8.981	10.251	8.611	8.909
	2	8.711	9.001	9.031	8.843
	3	8.838	7.278	8.888	8.778
	Delta	0.270	2.973	0.420	0.130
	Rank	3	1	2	4

**Table 7:** ANOVA for ultimate tensile strength and elastic modulus.

Response	Source	DF	SS	MS	F-Value	*p*-Value	Contribution (%)
UTS (MPa)	Layer thickness	2	15.420	7.710	6.450	0.008	0.29
	Build orientation	2	5192.750	2596.380	2172.900	0.000	98.16
	Raster angle	2	31.630	15.820	13.240	0.000	0.60
	Extrusion temperature	2	28.760	14.380	12.040	0.000	0.54
	Error	18	21.510	1.190			0.41
	Total	26	5290.080				100
Elastic modulus (GPa)	Layer thickness	2	0.030	0.015	2.560	0.105	0.72
	Build orientation	2	3.954	1.977	338.520	0.000	94.83
	Raster angle	2	0.073	0.037	6.280	0.009	1.76
	Extrusion temperature	2	0.007	0.004	0.610	0.554	0.17
	Error	18	0.105	0.006			2.52
	Total	26	4.170				100

DF is degrees of freedom; Adj SS is the adjusted sum of squares; Adj MS is the adjusted mean square.

**Table 8:** Confirmation of Taguchi method and linear regression equation.

Response	Optimum Level	Taguchi Method			Linear Regression Equation		
		Experimental	Predicted	Error	Experimental	Predicted	Error
UTS	L1B1R2T1	53.53 MPa	55.85 MPa	4.33%	53.53 MPa	55.11 MPa	2.95%
Elastic modulus	L1B1R2T1	3.28 GPa	3.37 GPa	2.74%	3.28 GPa	3.36 GPa	2.44%

## Data Availability

The original contributions presented in this study are included in the article. Further inquiries can be directed to the corresponding author.

## Appendix A

**Table A1:** Result of tensile test.

Run	UTS (MPa)				Elastic Modulus (GPa)			
	S1	S2	S3	AVE ± SD	S1	S2	S3	AVE ± SD
1	52.94	53.42	53.38	53.25 ± 0.27	3.21	3.26	3.26	3.24 ± 0.03
2	45.50	45.42	42.79	44.57 ± 1.54	2.96	2.85	2.97	2.93 ± 0.06
3	21.17	18.90	19.31	19.79 ± 1.21	2.49	2.37	2.19	2.35 ± 0.15
4	51.12	52.87	51.22	51.74 ± 0.98	3.23	3.29	3.23	3.25 ± 0.04
5	43.12	44.95	43.82	43.96 ± 0.92	2.80	2.85	2.79	2.81 ± 0.03
6	17.02	15.80	16.40	16.41 ± 0.61	2.25	2.13	2.28	2.22 ± 0.08
7	50.65	53.23	51.42	51.77 ± 1.32	3.23	3.31	3.26	3.27 ± 0.04
8	41.01	41.19	39.74	40.65 ± 0.79	2.75	2.71	2.71	2.72 ± 0.03
9	20.13	23.06	22.21	21.80 ± 1.51	2.25	2.43	2.46	2.38 ± 0.11

S1 is the first replica of the tested specimen; S2 is the second replica; and S3 is the third replica; AVE is the average; SD denotes standard deviation.
